# Experiences and barriers to implementation of clinical practice guideline for depression in Korea

**DOI:** 10.1186/1471-244X-13-150

**Published:** 2013-05-27

**Authors:** Jaewon Yang, Changsu Han, Ho-Kyoung Yoon, Chi-Un Pae, Min-Jeong Kim, Sun-Young Park, Jeonghoon Ahn

**Affiliations:** 1 Department of Psychiatry, Korea University College of Medicine, Seoul, South Korea; 2 Department of Psychiatry, Bucheon St. Mary's Hospital, The Catholic University of Korea College of Medicine, Bucheon, Kyounggi-Do, South Korea; 3 National Evidence-based Healthcare Collaborating Agency, Seoul, South Korea; 4 School of Pharmacy, Sungkyunkwan University, Suwon, South Korea; 5 Department of Psychiatry, Korea University Ansan Hospital, Korea University College of Medicine, 516, Gojan-dong, Danwon-gu, Ansan-si, Gyeonggi-do 425-707, South Korea

**Keywords:** Depressive disorder, Practice guidelines, Health care surveys, Questionnaires

## Abstract

**Background:**

Clinical guidelines can improve health-care delivery, but there are a number of challenges in adopting and implementing the current practice guidelines for depression. The aim of this study was to determine clinical experiences and perceived barriers to the implementation of these guidelines in psychiatric care.

**Methods:**

A web-based survey was conducted with 386 psychiatric specialists to inquire about experiences and attitudes related to the depression guidelines and barriers influencing the use of the guidelines. Quantitative data were analyzed, and qualitative data were transcribed and coded manually.

**Results:**

Almost three quarters of the psychiatrists (74.6%) were aware of the clinical guidelines for depression, and over half of participants (55.7%) had had clinical experiences with the guidelines in practice. The main reported advantages of the guidelines were that they helped in clinical decision making and provided informative resources for the patients and their caregivers. Despite this, some psychiatrists were making treatment decisions that were not in accordance with the depression guidelines. Lack of knowledge was the main obstacle to the implementation of guidelines assessed by the psychiatrists. Other complaints addressed difficulties in accessing the guidelines, lack of support for mental health services, and general attitudes toward guideline necessity. Overall, the responses suggested that adding a summary booklet, providing teaching sessions, and improving guidance delivery systems could be effective tools for increasing depression guideline usage.

**Conclusion:**

Individual barriers, such as lack of awareness and lack of familiarity, and external barriers, such as the supplying system, can affect whether physicians’ implement the guidelines for the treatment of depression in Korea. These findings suggest that further medical education to disseminate guidelines contents could improve public health for depression.

## Background

Depression is an enormous health-care problem that is responsible for 11% of disability worldwide [1]. It affects the quality of life and functioning of individual patients, and its high prevalence and substantial disease burden have significant societal and economic implications. The World Health Organization predicts that by 2020, major depression will be second only to ischemic heart disease as a cause of lost disability-adjusted life-years and untimely death [2].

For practicing psychiatrists, the guidelines provide many suggestions for different forms of treatment of the many kinds of depressive patients [3]. Clinical practice guidelines are “systematically developed statements to assist practitioners’ and patients’ decisions about appropriate health care for specific clinical circumstances” [4], and they aim to incorporate research findings and evidence-based practice for significant and consistent improvements in health care [5]. Their successful implementation may lead to improved quality of care by decreasing inappropriate variation in clinical practice and expediting the application of effective advances to everyday practice [6]. Increasing efforts are being undertaken to translate guidelines into clinical practice [7-10]; however, the implementation of guidelines is a complex process influenced by many factors, such as the behavior of the physician, the guidelines themselves, and the way they are implemented [6,7].

By understanding which factors are associated with implementation of evidence-based guidelines for the management of depressive disorders, strategies can be identified to improve physician guideline adherence and to adapt and focus guidelines and thus improve the quality of depression care. There are numerous possible barriers to implementation, from the distribution of guidelines to the use of guidelines in practice by physicians. The literature on determinants of implementation of evidence-based guidelines on depressive disorders has primarily focused on socio-demographic patient characteristics and disease-related factors, such as patient age and severity of the disorder [11,12]. Despite the guidelines’ potential importance, few studies have examined the influence of professional- and practice-related factors on implementation of the depression guidelines. Physician adherence to guidelines may be hindered by a variety of barriers. Studies identified barriers to guideline implementation as including several physicians’ characteristics: lack of awareness of the guidelines, disagreement with the guidelines, insufficient self-efficacy for change, and negative attitudes towards guidelines in general (“cookbook medicine”), and inertia associated with faith in existing treatment practices. In addition, external barriers such as lack of time, lack of availability, and insufficient support by the organization were also listed [6].

In Korea, since the first Korean Medication Algorithm for Major Depressive Disorder was developed [13], updated practice guidelines have been provided in 2008 and 2010. The guidelines are a good reference to assist the treatment decisions of clinicians who treat major depressive disorder patients in Korea. Barriers to using the guidelines are thus important to identify, but the few studies investigating these barriers have had limitations, such as that data is scarce for Korean populations, one study have addressed clinical practice, priority has been given to the identification of the most effective and optimal treatments rather than the most economic ones, and the Korean health insurance regulations are strict [13].

We hypothesized that the provision of guideline-recommended care is influenced by characteristics of both the physician and the practice. Understanding experiences and barriers is important for the development of effective implementation strategies. The present study, which was initiated as a quality improvement project, aims to assess the feasibility of the depression guidelines for clinical settings in Korea. In this article, we investigate barriers to the implementation of the guidelines for managing depressive disorders. We used a questionnaire to ask staff about their knowledge and use of these guidelines. Our findings can help developers of guidelines, practice directors, and health-care services researchers to design effective interventions to change physician practice.

## Methods

### Participants & recruitment

**Figure 1:**
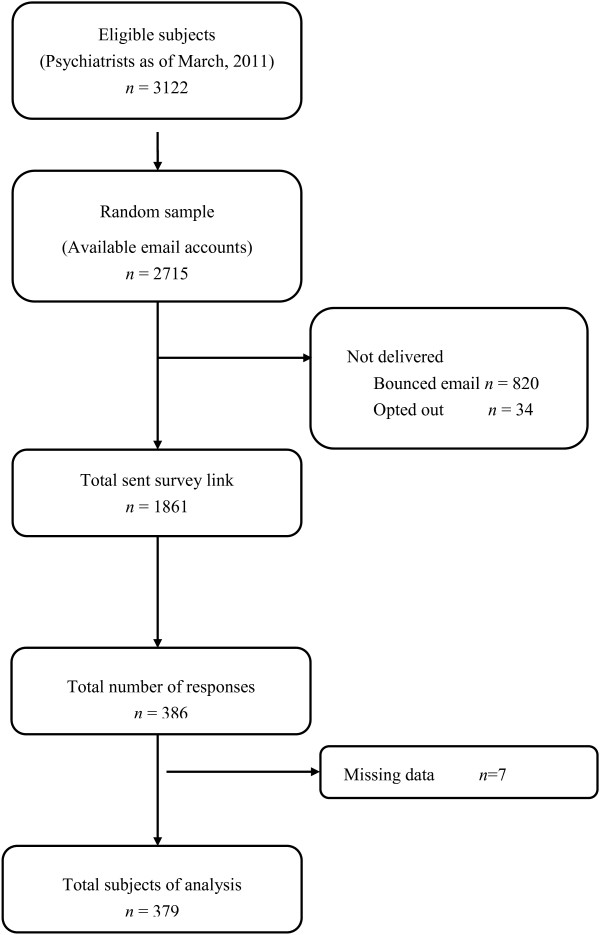
Disposition of survey participants.

This survey was performed from August 2011 to January 2012. To recruit expert participants for this study’s cross-sectional, web-based survey measuring met and unmet care needs, we used a modified Dillman method [14] which can help to maximize the response rate [15]. First, potential eligible participants were emailed a brief notice informing them about the study with an invitation to participate. This mailing list was generated from the email accounts in the annual report of the Korean Neuropsychiatric Association. Respondents could opt out of the study by clicking on an embedded URL link that terminated any further contact. The email message described the study in detail and included a hyperlink to the questionnaire. The invitation also offered a small incentive for participation (two coffee coupons). For non-responders, a reminder email was sent 10 days later, and a final email invitation was sent 10 days after that. The survey data collection was closed two weeks after the final email. Survey data, which were downloaded from the host, did not include respondents’ identifying information. The response rate was 14.2%. The recruitment and study flow chart is shown in Figure 1.

### Survey development

Questionnaire constructs for measuring the participants’ knowledge, experiences, attitudes, and barriers to implementation of the depression guidelines were developed from previous literature. The questionnaire contained three parts. The first part included demographic data for the psychiatrist: age, gender, clinical experience, employment status, and percentage of patients with depressive disorders. In addition, this part of the questionnaire contained items that assessed factors affecting diagnosing and treating depression. In part 2, we asked about the participants’ experiences with and attitudes toward the depression guidelines. This part contained items measuring perceived barriers to health care provision for patients with depressive disorders in terms of implementation of the evidence-based depression guidelines. Questionnaires also addressed the unmet needs in the depression guidelines. The study design was evaluated and approved by the Medical Ethics Review Committee of Korea University.

### Data analysis

Descriptive statistics are presented with frequency and percentage distributions for categorical data and means and standard deviations for continuous data. Chi-squared and independent sample t-tests, paired sample t-tests, and one-way ANOVA were employed for between-group comparisons. For all analyses, the level of statistical significance was set at *P* < 0.05. All statistical procedures were performed using SPSS software version 18.0 0 (SPSS Inc., Chicago, IL, USA).

## Results

### Participants’ characteristics

The descriptive statistics of selected characteristics of the participants are given in Table 1. The respondents comprised 277 (73.1%) men and 102 (26.9%) women, with a mean age of 41.7 ± 8.5 years. Forty-seven percent of the participants had psychiatric clinical experience over ten years. The mean percentage of patients with depression was 38.72% of the patients that the psychiatrist meets in one day.

**Table 1:** Descriptive Characteristic of participants (n = 379)

		**Male**		**Female**		**Total**	
		**(n = 277)**		**(n = 102)**		**(n = 379)**	
		**n**	**%**	**n**	**%**	**n**	**%**
Age (years)	30- 39	112	40.4	57	55.8	169	44.6
	40- 49	117	42.2	32	31.4	149	39.3
	50 - 59	37	13.4	7	6.9	44	11.6
	Over 60	11	4.0	6	5.9	17	4.5
Education	Bachelor	57	20.6	34	33.3	91	24.0
	Master	110	39.7	43	42.2	153	40.4
	Doctor	110	39.7	25	24.5	135	35.6
Employment status	Private practice	64	23.1	20	19.6	84	22.2
	Paid employment	74	26.7	38	37.2	112	29.5
	Military	17	6.1	0	0	17	4.5
	University hospital	92	33.2	31	35.2	123	32.4
	Mental hospital	30	10.8	13	12.7	43	11.3
Clinical experience	0 ~ 3 years	25	9.0	26	25.5	51	13.5
	3 ~ 5 years	36	13.0	21	20.6	57	15.0
	5 ~ 10 years	69	24.9	25	24.5	94	24.8
	Over 10 years	147	53.1	30	29.4	177	46.7

* *P* <0.05, chi-square test or Fisher's exact test.

Identified factors reported to affect diagnosis and treatment decisions were experience of the clinician in 258 (68.1%), treatment guideline in 107 (28.2%), and others such as textbooks, articles, recent reports, web-site information, inertia of previous practice, and other things in 14 (3.8%).

### Experiences and attitudes toward the depression guidelines

Seventy-five percent (n = 283) of psychiatrists reported that they had heard about the depression guidelines. Of these, 74.6% said they had read the depression guidelines. They first heard about the guideline through various sources: formal material from the academy (74.9%), their own reading of articles (11.9%), colleague discussions (4.7%), and the Internet and others (8.6%). The most recent time of referring to the guideline was reported as within the last 6 months in 46.0%, 6 to 12 months in 24.2%, and 12 months in 29.2% of participants. About 90 of the 211 (89.6% of) respondents who said they had read the guidelines also reported applying the depression guidelines in their practice, although most of them said they only applied the guidelines partially. Only 4.7% of psychiatrists reported using the guidelines exactly as written, and 5.7% of psychiatrists reported not using the guidelines although they had read them. About 90% of experts reported feeling slightly or moderately confident in using the guidelines, but only 10% were very confident in using them.

Approximately half (48.2%) of the experts reported using the guidelines some or most of the time when making decisions about patient care, while 26.8% reported using the guidelines for decisions about medication. Thus, about 73.9% of experts used the depression guidelines to some degree in their clinical practice. Other ways of using the guidelines included: for education of patients and their caregivers (19.4%), training of other clinical team members (15.79%), understanding depression in general, and diagnosis of depression. The mean rating of helpfulness of the guidelines on a visual analog scale was 50.65, and the factor most associated with assessing the treatment guidelines as reliable was the reliability to the organization that developed the guideline (57.0%).

### Barriers and unmet needs when employing the depression guidelines

A large proportion of the surveyed experts (88.1%) reported an opinion to recommend the depression treatment guidelines for team members. The reported reasons for recommending the guidelines were their usefulness for the treatment of depression (74.6%) and for agreement of treatment among doctors (24.3%). On the other hand, reasons for objecting to the guidelines were that it can be a limitation for clinical practice (42.2%), the difference between practice and the guidelines (24.4%), and questioning the usefulness of guidelines in general (20.2%) (Table 2).

**Table 2:** Willingness to use of depression treatment guidelines and the reasons of the responses

**Would you recommend the depression treatment guideline to your colleagues?**	**N**	**%**	**Reasons of the responses**	**N**	**%**
Yes	334	88.1	Usefulness of guideline for treatment	249	74.6
			Agreement between doctors	81	24.3
			Others	4	1.2
No	45	11.9	Questioning the usefulness of guideline	9	20.2
			Difference between practice and the guideline	11	24.4
			Limitation of using guideline in practice	19	42.2
			Others	6	13.2

**Figure 2:**
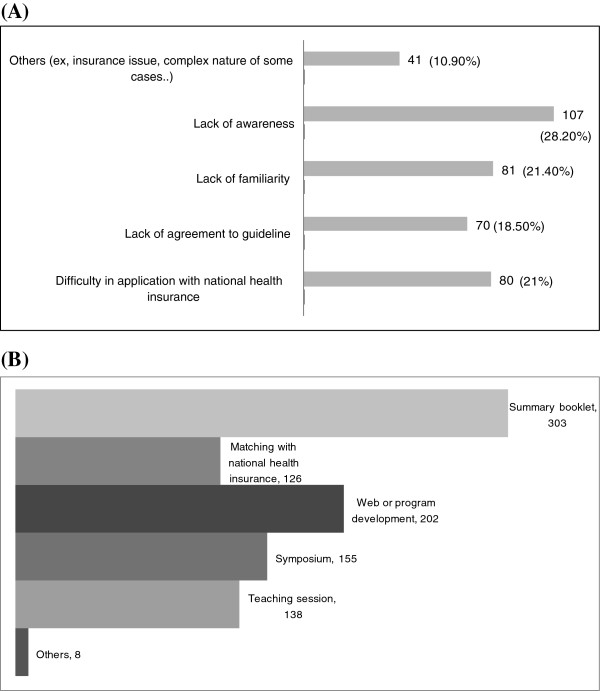
Barriers to use of depression treatment guidelines (A) and strategies for expanding the dissemination of depression treatment guidelines (B).

Participants reported that the barriers most related to implementing the depression treatment guidelines (with up to 3 selections allowed) were knowledge-related, such as lack of awareness (28.2%) and lack of familiarity (21.4%), attitude-related such as disagreement with guidelines and not concerning for experts (18.5%), and systems-related, such as lack of support from the government and difficulty in application with the national health insurance (21.0%). Other reasons included other insurance issues, patients’ misperception of depression and its treatment, the complex nature of some cases for which the guidelines provide no advice, not enough knowledge and understanding of the guidelines, and rejection of the treatments (10.9%). The experts asked for more help in using the depression treatment guidelines, and they stated the usefulness of a summary booklet, web-site, program development, teaching sessions or symposium, and compatibility with national health insurance (Figure 2). The unmet needs in the guidelines were indicated to be the need for better pharmacological treatments (33.6%), non-and pharmacological treatments (26.2%), and maintenance treatments (13.8%), as well as improved diagnosis for the disease (13.5%) and laboratory/scale tools for the disease (10.3%).

## Discussion

The aim of this study was to assess the experiences and barriers to physicians’ implementing Korea’s depression guidelines. Three quarters of psychiatrists reported having read the depression guidelines, and the majority of them said they applied the guideline in their practice at least partly. They tended to use the guidelines for making general decisions in clinical practice and for selecting medications. These experts also indicated that the guidelines were useful for educating patients and their caregivers. In a recent review of the difficulties associated with implementing guidelines, researchers suggested that even when practitioners agree with guidelines, they may not entirely implement them [16]. Additionally, it is know that there is a gap between evidence and practice in health-care research [17]. Some of the surveyed experts in the current study considered the depression guidelines to be important, but they indicated that they were not experienced in application and utilization of the guidelines. Nonetheless, our results suggest that the depression guidelines often facilitate decision making in critical clinical situations, as well as fostering informative communication with patients, caregivers, and the general population.

Even though that depression is treated by general practitioners in many countries, it is difficult for general practitioners (family physicians, internal medicine practitioners in Korea) to continue prescribing antidepressants more than two months because of the restrictions of reimbursement by the national Health Insurance Review and Assessment Service. Hence, depression patients are referred to psychiatrists when they are acknowledged by the general practitioners.

Most psychiatrists in this study reported intending to use the guidelines, but some were concerned about certain inherent limitations. Reasons for reluctance to use the guidelines included the following reported opinions: using the guidelines limits their professional independence, there is a gap between practice and guidelines, and using guidelines does not improve their work. Some were skeptical about the objectivity of guidelines and considered that recommendations could not be applied to their individual patients. Moreover, the most often reported barrier for implementation of the guidelines was little knowledge and experience with them. Health system restrictions were the next more reported barrier. Previous reports have also identified barriers at the clinician, clinical, and service levels [6].

It is important to be aware of these barriers so that they may be overcome. One essential factor in successful implementation is physicians' faith in the guidelines, particularly reflected in the common physician concerns about the relevance of guideline for patients and the loss of autonomy in treatment decision making. Acceptable, built-in, guideline-based treatment alternatives – rather than a single treatment mandate – would increase adherence by permitting physicians some level of treatment choice. Guidelines must also be easy to use and understand, as well as time efficient, in order to be sustainable [18,19]. Moreover, consideration of the health system is also needed at the service level. In this study, many psychiatrists responded that the guidelines are difficult to use due to strict national health insurance regulations and denial of individual health insurance use with the psychiatric record. Previous research has found that major obstacles for implementation of guidelines are time limitations (38.2%) and differences between guidelines and health insurance regulations (23.5%), in Korean studies of medication algorithms for bipolar disorder [20]. It is now recommended that implementation of guidelines should not be undertaken without addressing organizational level support.

Previous studies showed a number of strategies to encourage the use of clinical practice guidelines, including multifaceted interventions involving audit and feedback of treatment practices, reminders about appropriate use of guidelines, local consensus processes in adoption of guidelines, and interactive educational meetings [19]. As our survey results show, not only the relevance of the guideline but also active education and public relations to promote the guidelines with summary booklet, web-site programs, or educational sessions are needed for enhancing the applicability of the depression guidelines. Half of the surveyed psychiatrists reported high levels of confidence in using the guidelines, but very few had received any formal training on them, and many experts stated they would like more support in using the guidelines. The application of clinical practice guidelines includes dissemination and implementation. Dissemination processes include recognition, comprehension, and changing attitudes, and implementation processes include clinicians’ behavior changes in clinical practice [5]. To accomplish the goals of improving health while making more efficient use of health-care resources, even greater attention must be paid to their creation, dissemination, adoption, and re-evaluation [21,22]. Moreover, educational interventions positively influence physicians’ behavior toward the use of guidelines [23].

There are some limitations in this study that should be acknowledged. First, the survey results may not be generalizable to all the psychiatrists who did not participate, although the response rate of 14.2% is similar with previous studies. The list of potential participants also could include retired psychiatrists. However, we could not reveal that who is retired or not on the list. The response rate might be higher if this is taken into account. Also, annual report of Korean neuropsychiatric Association does not give information on the members’ education, and other demographic data. Authors assumed that the psychiatrists who did not answer would have similar educational background but there could be other issues in generalization of the present results. Clinician have poor responses rates to surveys in general [24], and web-based surveys may have lower response rates than telephone or mailed surveys [25]. Previous survey studies using web-based methods with a randomly selected sample reported similar results with the present study [26,27]. A second limitation is that we used a cross-sectional sampling method; it is possible that further conclusions could have been drawn from longitudinal data.

## Conclusions

This study has demonstrated that psychiatric experts providing mental health services frequently applied the depression guidelines for general decision making, for medication selections, and for information transmission to patients and their caregivers. Physicians’ adherence is critical in translating evidence-based recommendations into improved outcomes. However, individual factors such as lack of awareness and lack of familiarity and external barriers such as the reimburse restriction can affect the employment of the guidelines in the real practice. Changes should be made to improve public health by increasing implementation of the guidelines, but proper implementation can be achieved with active promotion of the guideline contents [16] and additional efforts for the improvement of the guidelines in evidence-based and also in expert-consensus manners.

## Competing interests

There are no conflicts of interest for any of the authors. All authors disclose any financial and personal relationships with other people or organizations that could inappropriately influence and/or bias their work.

## Authors’ contributions

CH was integral in the design and writing of the article. JY conducted analyses and wrote the first draft of the manuscript. S-YP and M-JK recruited the subjects and performed the web-survey. H-KY, C-UP and JA contributed to the interpretation of data and gave their advice throughout the study. All authors contributed to and have approved the final manuscript.

## Pre-publication history

The pre-publication history for this paper can be accessed here:

http://www.biomedcentral.com/1471-244X/13/150/prepub
